# Effects of orthoses on muscle activity and synergy during gait

**DOI:** 10.1371/journal.pone.0281541

**Published:** 2023-02-09

**Authors:** Yu Hashiguchi, Ryosuke Goto, Toru Naka

**Affiliations:** 1 Department of Physical Therapy, Faculty of Rehabilitation, Gunma PAZ University, Takasaki-shi, Gunma, Japan; 2 Department of Speech-Language-Hearing Therapy, Faculty of Rehabilitation, Gunma PAZ University, Takasaki-shi, Gunma, Japan; 3 Department of Physical Therapy, Faculty of Rehabilitation, R Professional University of Rehabilitation, Tsuchiura-shi, Ibaraki, Japan; Ningbo University, CHINA

## Abstract

An orthosis is often used in rehabilitation to improve kinetic and kinematic parameters during gait. However, whether changes in neural control depend on wearing an orthosis during gait is unclear. We measured the muscle activity and synergy of the lower limb muscles without orthosis and with two types of orthoses: ankle–foot orthosis (AFO) and knee–ankle–foot orthosis (KAFO). Muscle activity during gait was measured in 15 healthy adults, and muscle synergies were extracted using non-negative matrix factorization. The results revealed that some muscle activities were significantly different among the three conditions. Post-hoc analysis indicated differences between each condition. Knee extensor muscle activity related to the loading response was significantly increased by wearing the AFO. In the KAFO condition, hip abductor muscle activity related to weight bearing was significantly decreased, and ankle dorsiflexor muscle activity was increased to secure clearance during the swing phase. However, the number of muscle synergies and complexity of muscle synergy did not significantly change among these conditions. However, along with changes in muscle activity, the activation pattern and weightings of muscle synergies tended to change with the use of orthoses. Each muscle activity was changed by wearing the orthosis; however, the immediate mechanical constraint did not change the framework of muscle synergy.

## Introduction

Orthoses are widely used in certain situations, including in clinical settings, sports, and daily living. In particular, lower limb orthosis is used during gait and restricted lower limb motion, which causes changes in kinetics, kinematics, gait parameters, energy cost, and sensation.

In healthy adults, restricted ankle motion resulting from an orthosis significantly reduces gait speed [1]. However, the energy cost during gait is improved by wearing an orthosis [2]. Furthermore, orthosis may change the sensory input and cause a change in the H-reflex and Hoffmann reflex; however, the results of these reports are inconsistent [3, 4].

Specifically, previous studies in healthy adults have reported the effect of an orthosis on biomechanics during gait. Romkes has reported that the wearing side of the pelvis was elevated because of restricted ankle motion by orthosis [1]. Furthermore, this study revealed that the hip was significantly less extended during the stance phase. In the swing phase, the hip was more adducted, and the peak hip flexion significantly increased and occurred earlier. Simultaneously, the knee flexion during the loading response significantly decreased, and the peak flexion during the swing phase significantly increased. On the other hand, Vistamehr has reported that the propulsion impulse was significantly decreased by an ankle–foot orthosis (AFO) [5]. And as the reason for this, there are a decrease in the peak ankle plantar flexor moment and an increase in the hip extensor moment. Kato has reported that the time of the stance phase was relatively shorter with AFO and that the trunk tilted forward during toe-off as a compensative for restricted ankle motion [6]. These results suggest that restricted joint motion due to wearing orthoses may cause various compensatory movements and change in muscle activities during gait.

In rehabilitation of patients with stroke, AFO and knee–ankle–foot orthosis (KAFO) are commonly used. Specifically, the AFO is used to fix the ankle joint, and the KAFO is used to fix the knee and ankle joints. Meta-analyses have revealed that the AFO changes the kinetics, kinematics, and energy cost during gait in patients with stroke [7]. Specifically, wearing the AFO altered the ankle angle at initial contact and peak dorsiflexion during the swing phase [8]. Additionally, the AFO altered the knee kinematics, including the knee flexion angle at initial contact at the loading response [8]. As parameters of kinetics, the trajectories of the center of pressure under soles during gait were also altered by AFO [9]. Similarly, in children with cerebral palsy, the AFO has been demonstrated to cause an increase of 34% in gait speed and 18% in stride length [10, 11].

KAFO is also used for muscle weakness of the knee joint related to the central nervous system and neuromuscular diseases, including stroke, spinal cord injury, and post-polio syndrome [12]. In healthy adults, wearing KAFO causes a reduction in gait speed and stride length and an increase in some muscle activities [13]. However, evidence of the effect of KAFO is limited.

Muscle activity is measured by surface electromyography to assess neutral control, and muscle synergy has recently been reported to demonstrate the characteristics of muscle coordination [14–17]. Muscle synergy demonstrates modular neural control of multiple muscles according to movement [14]. Few muscle synergies during gait have been demonstrated, which may be related to the central pattern generator (CPG) [16]. Previous research has also reported the characteristics and changes in muscle synergy in several patients, from newborn babies to adults [15, 17, 18]. In patients with stroke and children with cerebral palsy, the number of muscle synergies is reduced and significantly related to motor function [19, 20]. Additionally, each muscle synergy corresponds to the mechanical demand [14]. For instance, previous research has reported that muscle synergies during gait correspond to body support in the early stance phase, trunk propulsion in the late stance phase, and leg deceleration in the swing phase [21]. From these facts, wearing of orthoses may limit the mechanical response during gait and may change muscle activity and muscle synergy. This study aimed to clarify the changes in muscle activity and synergy during gait with and without orthosis in healthy adults.

Previous studies have revealed that the powered ankle exoskeleton did not change the number of muscle synergies but changed the weightings and activity pattern of muscle synergy [22]. In this study, we hypothesized that the modularity of the muscle synergy may not change. However, the change in the behavior of muscle synergy, including weightings and activity pattern muscle activity would occur relating to the change in muscle activity due to orthosis wearing.

## Materials and methods

### Participants

A convenience sample of 15 healthy, male students from Gunma Paz University (mean±SD: age 20.60 ± 0.61 years; height 172.37 ± 3.30 cm; weight 66.04 ± 10.10 kg) was recruited. Participants who had orthopedic disease within 6 months were excluded. All participants provided written informed consent. This study was conducted in accordance with the Declaration of Helsinki and approved by the Research Ethics Review Board of Gunma Paz University (PAZ18-32).

### Procedure

The participants were made to walk on the treadmill at a comfortable speed. A Trigno wireless system (Delsys Co., Boston, USA; sampling rate: 2000-Hz) was used to record the activity of eight lower limb muscles. The recorded muscles, including the rectus femoris (RF), vastus medialis (VM), gluteus medius (GM), biceps femoris (BF), semitendinous (ST), tibialis anterior (TA), lateral gastrocnemius (GS), and soleus muscles (SL), and electrodes were attached to the left leg in accordance with SENIAM [23]. For preparation, the skin surface was treated with alcohol, and the electrodes were attached using special double-sided tape and surgical tape. In addition, an elastic belt was used to decrease the collision noise with the brace. An additional sensor, which included a triaxial accelerometer synchronized with the electromyography (EMG), was attached to the posterior surface of the shoe part of orthosis, which is close to the heel, to identify heel contact.

**Fig 1:**
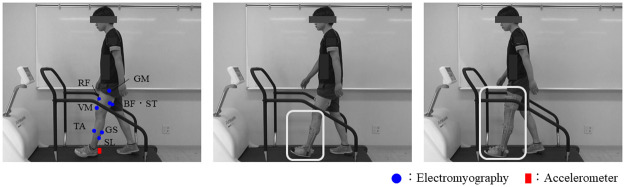
Measurement of the three conditions. Blue circles and red squares show the positions of the electromyography and accelerometer, respectively. An accelerometer was attached to the posterior surface of the shoe to detect heel contact during gait.

The following experimental conditions were randomly conducted: walking without orthosis (Normal), walking with an AFO (AFO), and walking with a KAFO (KAFO) (Fig 1). The AFO and KAFO were selected from two types, medium and large, and all participants wore the orthosis on the left lower limb for measurement. A practice session was performed on the ground level before the measurement. During the practice, the participants wore the two orthoses of medium and large sizes, and the appropriate size and the pain due to wearing the orthoses was assessed by questioning the participant. If the participant complained of pain while walking on level ground, the measurement was stopped. All measurements were performed on a treadmill. Hence, the Normal and AFO conditions were conducted in all the participants. Of the 15 participants, 12 completed the KAFO condition, and these data were used for the following analysis.

Before the recording session, the participants walked on a treadmill without an orthosis and determined the comfortable speed. At the recording session, the treadmill was started with the participants standing on the belt, and the speed was gradually increased to a comfortable speed. The recording was conducted for 3 min after the participants walked for 2 min to settle into a regular pattern.

### Data analysis

First, the average and maximum muscle activities of each muscle were calculated. The corrected data were high-pass filtered (40 Hz), rectified, and low-pass filtered (10 Hz) [22]. The heel contact time was identified using vertical acceleration data with reference to a previous study [24]. First, the gait cycle was detected, in particular, the stance phase, which demonstrated little variability, and the swing phase, which indicated gradual and large variability. Second, heel contact was identified from steep spikes that occurred after the swing phase. Based on the time of heel contact identified by the accelerometer, the EMG data were extracted for five gait cycles, normalizing the time for each gait cycle, and the average waveform was obtained from five gait cycles. The initial outcomes of the average muscle activity (AEMG) and maximum muscle activity (MEMG) were calculated from the averaged waveforms.

Second, the outcome of the muscle synergy was calculated. The number of synergies and the dynamic motor control index during walking (DMC) was calculated as the outcomes of muscle synergies, which reflect the complexity of muscle coordination [25]. To improve the analysis speed, the EMG data were down-sampled at 50-m bins, and the data for 1 min, the middle of the 3-min measurement, was used for analysis. The non-negative matrix factorization (NNMF) algorithm extracted muscle synergies using MATLAB R2019b. This method calculates the two matrices Ci(t), which denotes the activation pattern of muscle synergies, and Wi, which represents the weighting of muscles involved in each synergy. The following equation represents the original EMG data *m[t]*:

$$m\left(t\right)=\;\sum C_{i}\left(t\right)W_{i}\left(t\right)+e$$ (1)

In this algorithm, the initial values of the two matrices were set randomly, and the appropriate matrices (C and W) were calculated by 100 iterations using a previously reported algorithm [26]. *e* indicates the difference between the raw data *m(t)* and reconstructed data *(Ci × Wi)*. The number of muscle synergies may be between 1 and 8, and the number of muscle synergies was obtained using the value of variability accounted for (VAF) [27]. The VAF indicates the percentage of variance explained between the raw and reconstructed EMG data. VAF is the ratio of the sum of the squared error (e) to the sum of the squared total data m (t).


$$VAF=\left\{1-e^{2}/m{\left(t\right)}^{2}\right\}\times 100\left(\%\right)$$ (2)

The number of synergies was obtained using the following two criteria from previous studies [28]. The first is the case where the total VAF (tVAF) exceeds 90%, with an amount of change of <5%, and the second is the case where the VAF of each muscle exceeds 75%, with an amount of change of <5%. These criteria using VAF ensured that the selected muscle synergies adequately reconstructed muscle activity in each condition. According to this threshold, four synergies accounted for the EMG activities under most conditions. The Cis of the four muscle synergies under normal conditions were arranged according to the weightings of the muscles. Muscle synergies in the AFO and KAFO conditions were sorted based on the correlation coefficient with the normal condition. The sorted muscle synergies were named in the following order: Syn1, Syn2, Syn3, and Syn4. Additionally, walk-DMC was calculated as the Z score of the VAF when the NNMF algorithm was conducted, assuming that the number of synergies was 1. Walk-DMC represents the complexity of the muscle activity during each condition.

### Statistical analysis

Statistical analyses were performed using SPSS ver28 (IBM, Armonk, NY, USA). First, a Shapiro–Wilk test was performed to check the normality of the parameters. The parameters of muscle activity (AEMG and MEMG) and muscle synergies (number of synergies and walk-DMC) were compared among the three conditions using the Friedman test, and the multiple comparison test with the Bonferroni method was used to identify significant differences between each post-hoc group. The significance level was set at p < 0.05.

## Results

**Fig 2:**
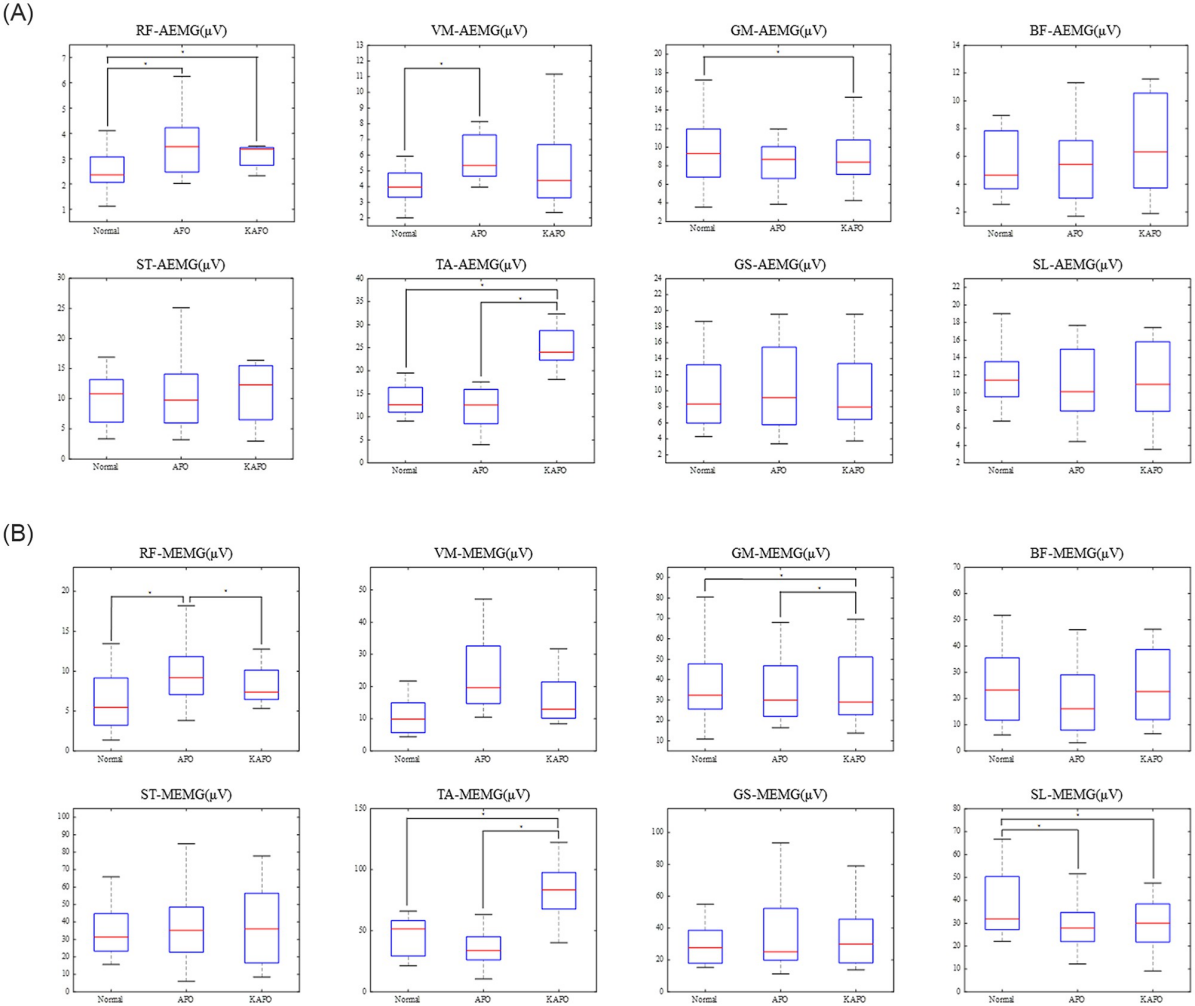
Results of the AEMG (2A) and MEMG (2B) of each muscle. Asterisks represent significant differences (p < 0.05) between each condition revealed by post hoc analysis.

For AEMG, significant differences were observed in RF, VM, GM, and TA (Table 1, Fig 2A). RF and VM were increased in the AFO compared to the Normal (p < 0.01, p = 0.01, respectively). GM was reduced in the KAFO compared to the Normal (p = 0.04). In the KAFO condition, TA was increased compared to the Normal and AFO conditions (p < 0.01, p < 0.01).

**Table 1:** Results of muscle activity and parameters of muscle synergies.

	Friedman test		Normal-AFO	Normal-KAFO	AFO-KAFO
	χ2	*p*	*p*		
AEMG (μV)					
RF	**12.67**	**<0.01** ^*^	**<0.01** ^**†**^	**0.01** ^**†**^	1.00
VM	**8.17**	**0.02** ^*^	**0.01** ^**†**^	0.46	0.46
GM	**6.50**	**0.04** ^*^	1.00	**0.04** ^**†**^	0.20
BF	2.67	0.26	–	–	–
ST	0.00	1.00	–	–	–
TA	**15.17**	**<0.01** ^*^	1.00	**<0.01** ^**†**^	**<0.01** ^**†**^
GS	0.50	0.78	–	–	–
SL	2.67	0.26	–	–	–
MEMG (μV)					
RF	**10.50**	**0.01** ^*^	**0.01** ^**†**^	1.00	**0.04** ^**†**^
VM	**6.17**	**0.05** ^*^	0.07	1.00	0.12
GM	**14.00**	**<0.01** ^*^	0.66	**<0.01** ^**†**^	**0.04** ^**†**^
BF	2.00	0.37	–	–	–
ST	1.17	0.56	–	–	–
TA	**15.17**	**<0.01** ^*^	1.00	**<0.01** ^**†**^	**<0.01** ^**†**^
GS	1.17	0.56	–	–	–
SL	**13.17**	**<0.01** ^*^	**0.02** ^**†**^	**<0.01** ^**†**^	1.00
Syn parameters					
Syn num	5.00	0.08	–	–	–
DMC	1.17	0.56	–	–	–
				*: p<0.05	†:p<0.05

The results of the Friedman test and post hoc analysis for the averaged and maximum EMG values. Asterisks represent significant differences (p < 0.05) among the three conditions using the Friedman test. Daggers indicate a significant difference (p < 0.05) between each condition revealed by post hoc analysis.

AFO, ankle–foot orthosis; KAFO, knee–ankle–foot orthosis; RF, rectus femoris; VM, vastus medialis; GM, gluteus medius; BF, biceps femoris; ST, semitendinous; TA, tibialis anterior; GS, gastrocnemius; SL, soleus muscles; AEMG, average muscle activity; MEMG, maximum muscle activity.

Regarding MEMG, significant differences were observed in the RF, VM, GM, TA, and SL (Table 1, Fig 2B). The RF was increased in the AFO compared to the Normal (p = 0.01). For the GM, the KAFO had a significantly lower value than the Normal (p < 0.01). In the lower leg muscles, the KAFO showed a higher TA activity than the Normal and AFO (both p < 0.01). In SL, for which no significant difference was observed in AEMG, the AFO and KAF condition showed significantly reduced MEMG than the Normal (p = 0.02 and p < 0.01, respectively).

**Fig 3:**
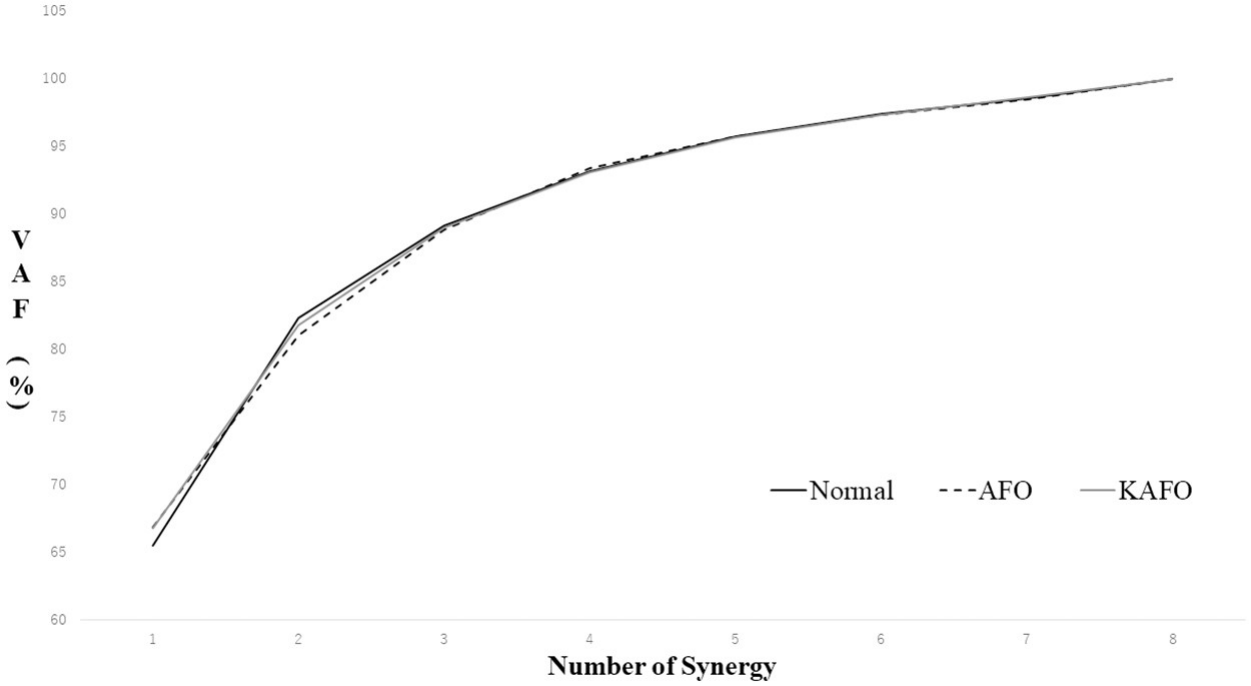
Total VAF among three conditions. The black solid line represents tVAF under Normal conditions, and the black dashed and gray solid lines indicate the AFO and KAFO conditions, respectively.

**Fig 4:**
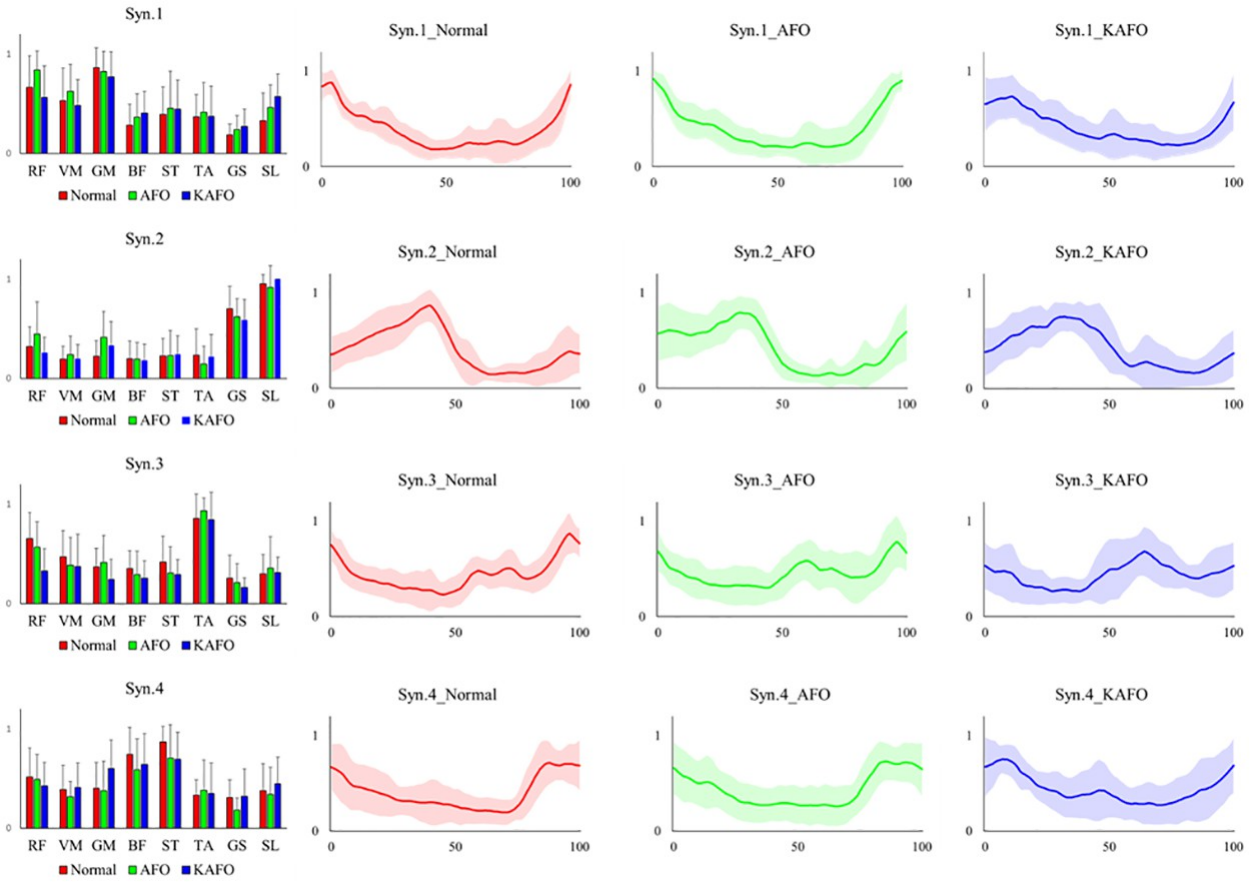
The characteristics of muscle synergy. The left figures represent the activation pattern of muscle synergies, and the right figures demonstrate the weighting of muscles involved in each synergy among the three conditions (Normal/AFO/KAFO).

Table 1 lists the results of the parameters of muscle synergies. Fig 3 presents the total VAF for the three conditions. Unlike the results of the single muscle activity, the Friedman test revealed no significant difference in the number of synergies and tVAF among the three conditions. In addition, no significant difference in DMC was observed among the three conditions. Fig 4 presents the weightings and average activities of the muscle synergies, which tended to change depending on the two orthoses. As for the activity of muscle synergy, the peaks of Syn1 and Syn4 tended to delay under the KAFO condition. In the AFO and KAFO conditions, the peak of Syn2 flattened compared to that in normal conditions. Additionally, the activity of Syn3 flattened throughout the gait cycle. As weightings of muscle synergies, in the KAFO condition, Syn3 was changed to include the lower limb muscles. In addition, the weightings of Syn4 flattened in the KAFO condition.

## Discussion

The results of the AEMG revealed that walking with the AFO caused an increase in knee extension muscle (RF and VM) activity, while walking with the KAFO caused a reduction in GM activity. TA activity increased in the KAFO condition. Moreover, for SL, only the maximum activity increased when the KAFO was worn. However, no significant difference in the value of the number of synergies and DMC was observed, although the activity of muscle synergy tended to change with muscle activity. The results suggest that the presence or absence of orthosis did not influence the modularity of muscle synergy, but changed the behavior of muscle synergy related to individual muscle activity.

RF and VM are responsible for eccentric contraction and propelling the thigh forward in a double-knee action during the loading response phase of walking [29] and related synergy 1 (Fig 4). In the simulation study, Yamamoto has demonstrated that wearing an AFO caused an increase in knee flexion angle and peak knee joint reaction force during the stance phase, and the quadricep muscle force was also significantly increased [30]. This change may occur because of the prevention of knee overflexion, and the increase in RF and VM muscle activity may occur due to this kinematic change when wearing orthoses.

The GM is thought to support weight and stabilize the lower limb during the mid-stance phase [29]. The ankle and knee joints were fixed using the KAFO, thus suppressing GM activity. However, TA activity was increased by wearing the KAFO. When a KAFO is worn, maintaining foot clearance during the swing phase may be challenging because of the restriction of the range of ankle dorsi-flexion and knee flexion. Thus, participants may increase their TA activity to avoid tripping. Similarly, SL, which is active during the pre-swing phase related to ankle dorsiflexion during the swing phase, may increase its activity in the KAFO condition. Furthermore, in this study, the measurement was performed on the treadmill. It is known that walking on a treadmill result in a more passive gait than walking on level ground. Especially, it is considered that the belt move might relate to the change in TA and SL activity to maintain the clearance.

Regarding the change in muscle synergy, the complexity of synergistic control, represented by the number of synergies and DMC, was not changed by the mechanical constraints owing to the orthosis.

Previous studies have demonstrated the robustness of muscle synergy during gait. Muscle synergy may be the learned modules of muscle activity resulting from the mechanical demand, and it is consistent for each movement. Ivananeko et al. have reported that when participants walked with another motion, such as kicking, which requires different mechanical demands, the gait-related muscle synergies are preserved, and additional muscle synergy is generated [15]. They have also revealed that in patients with spinal cord injury, whose injury were mainly at thoracic level, multiple muscle synergies were maintained [31] and it is considered that muscle synergies during gait may be generated by CPGs in the lower spinal cord. Our results may also provide important implications regarding the robustness by which muscle synergies respond to immediate mechanical limitations. Here, the muscle synergies were fine-tuned in the activation pattern and weightings; however, the organization was preserved even under mechanical limitations. Thus, muscle synergies may be constant and centrally controlled. These results were similar to those of Jacobs, who has reported on the effects of a powered exoskeleton on muscle synergy in healthy adults. The study has demonstrated that the organization (module size and function) of muscle synergy was preserved, although changes occurred in activation pattern rather than in weightings [32].

On the other hand, Previous studies have also demonstrated changes in the organization of muscle synergies [19, 20, 33, 34]. Patients after stroke reportedly have a decreased number of synergies during gait than healthy adults [19]; however, another study has reported that the number of synergies did not change in patients after stroke [33]. Additionally, the number of synergies in children with cerebral palsy has been consistently reduced in previous studies [20]. In addition, Kargo has reported that muscle synergy is changed by skill learning [34]. Hence, the change in muscle synergy does not occur instantaneously but occurs due to long-term effects related to motor disorders and motor learning. However, along with changes in muscle activity, the activation pattern and weightings of muscle synergies may change depending on the use of orthoses in this study. Future studies should investigate these parameters as a characteristic of muscle coordination.

This study had some limitations. First, the participants were healthy adults. Orthoses are widely used in rehabilitation, and their effects have been demonstrated in patients with certain diseases such as stroke, cerebral palsy, and spinal cord injury. To apply the results of this study, the effect of orthoses on muscle activity and muscle synergy in patients with each disease need to be clarified. Second, the sample size was small, and the data for the three participants were lacking because of pain while wearing the KAFO. The number of participants was 20% of the total number. Thus, further studies with a larger sample size are required to demonstrate the effects of KAFO. Furthermore, the reason for the uncomfortableness with KAFO may be caused by the size of the orthosis. Specifically, in AFO conditions, the measurements were performed without pain by adjusting the length of the belt for differences in lower leg circumstances. However, the three participants, who wore the KAFO for measurements, complained of pain mainly from tightness in the thigh, which was thought to be caused by excessive pressure with insufficient belt length, and we decided to avoid performing their measurements. The choice of orthosis sizes for all subjects is considered difficult, and this problem is likely to occur in a clinical situation. Recently, new orthoses which have changeable sizes for various subjects have been developed and they should be used in the future studies. Third, this study did not analyze kinematic parameters, and the measurement of kinetic and kinematic parameters is required to account for the background of changes in muscle activity. Last, measurements were conducted at a comfortable speed. The change in gait speed may have affected the results, and patients in clinical settings may exhibit various gait speeds. Thus, the effect of gait speed should also be considered. The novel results of this preliminary study may be useful for future research, although for the clinical application of orthosis, future studies with a larger sample size should investigate various conditions, including gait speed, to demonstrate orthosis-related changes in muscle activity and synergies in patients.

## Conclusion

We investigated muscle activity changes and muscle synergies in healthy adults wearing AFO and KAFO. Each muscle activity changed when wearing an orthosis, although the framework of muscle synergy was not changed by the immediate mechanical constraint.

## Supporting information

S1 Table Minimal dataset. The value of parameters in each participant.
(PDF)
