# Supplementary material for: Effects of orthoses on muscle activity and synergy during gait
Source: PLoS One. 2023 Feb 9;18(2):e0281541. doi: 10.1371/journal.pone.0281541 (PMC9910715; doi:10.1371/journal.pone.0281541)
Supplement: S1 Table — The value of parameters in each participant. (PDF) [file pone.0281541.s001.pdf]

Supporting information-Minimal dataset

Parameter: AEMG (μV)

Condition: Normal

ID	RF	ML	GM	BF	ST	TA	GS	SL
1	2.62	4.78	8.60	3.67	6.43	12.62	12.72	13.63
2	3.07	2.60	17.21	15.06	16.89	12.01	15.66	23.58
3	4.11	4.93	6.50	3.22	11.09	10.74	6.31	10.42
4	2.29	3.72	5.16	7.42	12.03	9.75	9.65	13.27
5	1.49	2.01	11.52	3.08	5.98	14.50	8.35	19.01
6	2.16	3.81	20.55	8.62	13.51	17.86	7.12	12.65
7	2.98	3.51	9.32	5.95	28.42	27.92	13.44	10.67
8	5.18	5.93	14.52	8.95	15.13	12.04	8.44	6.75
9	2.36	4.45	7.62	4.66	12.23	13.97	18.64	11.44
10	2.03	5.42	9.69	4.12	5.82	9.07	5.81	9.36
11	1.11	2.20	8.45	7.98	3.34	9.42	4.28	8.52
12	2.34	4.46	5.37	3.72	5.09	19.52	5.86	18.12
13	1.77	3.25	3.53	2.53	8.14	11.81	14.67	10.06
14	3.08	3.97	9.36	4.59	10.85	17.03	6.91	7.62
15	3.07	4.90	12.09	7.18	9.31	14.38	4.82	12.91

Parameter: AEMG (μV)

Condition: AFO

ID	RF	ML	GM	BF	ST	TA	GS	SL
1	3.81	8.14	8.68	2.84	5.37	11.10	9.80	10.11
2	6.25	11.65	23.43	9.63	15.46	13.30	19.54	26.62
3	2.02	3.97	6.26	3.45	8.71	6.15	7.13	7.67
4	5.33	7.74	6.64	11.31	9.79	7.87	17.46	15.61
5	4.30	5.10	9.96	4.54	12.58	10.54	13.81	17.67
6	3.99	11.42	15.36	6.42	19.53	17.58	7.25	15.05
7	3.08	5.08	6.63	5.48	25.10	40.28	16.00	9.02
8	4.37	5.33	10.06	4.84	10.75	10.84	3.48	4.64
9	2.51	4.38	8.89	5.80	9.57	16.21	17.25	10.65
10	2.49	5.92	9.71	7.37	10.29	12.54	11.48	10.11
11	2.29	5.58	8.44	2.63	3.47	4.66	5.44	8.86
12	2.46	4.52	5.11	5.42	6.12	31.57	3.37	8.68
13	2.06	4.20	3.86	1.68	3.21	3.96	5.42	4.42
14	3.77	5.32	8.50	2.34	5.93	12.83	6.76	5.84
15	3.47	5.76	11.94	9.18	14.56	15.27	9.12	14.66

Parameter: AEMG (μV)

Condition: KAFO

	RF	ML	GM	BF	ST	TA	GS	SL
1	4.63	8.83	8.55	3.94	8.12	23.36	9.71	10.81
2	3.39	5.88	20.28	11.15	11.93	24.38	14.08	17.14
3								
4								
5	2.58	2.36	9.29	3.49	4.91	21.24	16.77	14.48
6	3.40	11.16	15.36	11.58	34.28	26.99	8.37	17.43
7	3.50	4.28	6.14	3.51	16.39	79.54	5.53	10.70
8	2.97	3.17	8.55	6.80	11.73	11.85	5.99	3.53
9	2.75	4.49	8.23	9.41	15.93	30.46	19.56	11.11
10	2.32	3.41	8.23	10.30	12.70	23.74	6.83	8.59
11	2.72	3.45	6.62	5.63	3.04	25.05	7.59	7.15
12	3.36	7.47	4.26	5.83	15.11	32.34	12.72	17.38
13								
14	3.47	2.89	7.50	1.89	2.97	18.14	3.75	4.14
15	3.38	5.60	12.25	10.81	13.49	23.63	7.53	13.74

Parameter: MEMG (μV)

Condition: Normal

ID	RF	ML	GM	BF	ST	TA	GS	SL
1	3.67	6.26	32.34	11.46	26.89	29.12	32.33	36.30
2	9.73	4.44	64.07	95.63	65.83	59.99	38.71	60.98
3	24.82	15.34	28.60	7.08	25.26	34.20	16.98	27.88
4	5.25	5.46	12.10	19.01	45.18	21.29	38.20	51.06
5	2.53	5.37	60.88	12.19	31.31	51.56	26.03	66.76
6	3.09	9.89	80.50	51.70	43.44	58.80	16.95	31.84
7	6.93	11.23	26.18	23.25	88.47	136.53	46.34	30.39
8	11.60	21.73	47.03	32.20	42.93	26.49	30.38	22.10
9	6.79	13.75	25.53	11.60	30.19	52.44	55.04	30.56
10	5.45	30.67	38.30	24.62	41.53	27.82	20.52	27.02
11	1.37	4.79	34.12	50.66	15.63	56.66	15.34	37.16
12	4.45	9.45	15.82	26.07	16.30	65.99	16.75	53.66
13	2.70	9.19	10.89	6.10	22.67	29.72	52.38	23.98
14	7.40	11.18	25.65	22.27	22.50	46.18	25.52	23.06
15	13.45	29.41	47.91	36.60	54.06	51.40	27.72	48.26

Parameter: MEMG (μV)
Condition: AFO

ID	RF	ML	GM	BF	ST	TA	GS	SL
1	10.89	29.95	30.55	7.79	35.20	33.76	22.81	25.98
2	18.20	47.11	62.43	46.22	50.63	44.31	67.36	61.97
3	3.82	10.52	27.57	7.91	23.34	16.04	25.00	25.30
4	21.35	35.12	20.11	31.51	28.00	33.45	93.41	51.58
5	11.81	24.26	53.31	15.66	59.84	26.06	30.61	48.06
6	9.18	36.30	67.96	27.33	48.78	63.30	17.49	30.71
7	7.65	14.22	16.45	23.73	84.74	95.29	60.81	21.97
8	11.84	16.41	35.68	16.11	48.09	34.15	11.25	12.21
9	6.91	13.52	31.08	15.60	22.41	43.79	59.47	29.77
10	6.60	29.06	29.61	29.61	47.43	33.27	30.59	22.03
11	7.53	19.66	29.95	7.96	11.25	18.76	20.03	27.86
12	7.84	15.22	17.09	26.19	26.08	102.37	11.76	29.05
13	5.14	14.54	17.69	3.11	5.94	10.53	19.77	20.00
14	11.02	17.49	29.66	5.37	16.40	26.31	26.78	16.18
15	13.41	33.52	50.43	36.58	46.52	45.51	25.10	36.07

Parameter: MEMG (μV)
Condition: KAFO

ID	RF	ML	GM	BF	ST	TA	GS	SL
1	10.35	20.31	28.61	12.71	19.73	73.42	27.59	32.22
2	6.96	19.37	62.04	46.38	38.70	84.88	42.55	45.38
3								
4								
5	6.43	8.55	56.30	14.62	13.39	81.76	78.91	41.39
6	5.51	31.70	69.54	43.13	77.81	100.28	24.16	35.47
7	7.78	10.37	14.99	9.96	68.42	318.55	13.83	24.12
8	8.89	9.95	30.14	24.75	48.33	40.18	16.83	9.10
9	6.45	14.42	23.25	20.49	33.69	122.30	58.49	27.85
10	5.33	10.71	29.27	34.28	38.37	95.13	19.50	21.18
11	12.76	11.55	27.96	27.02	8.48	89.02	48.62	22.41
12	6.67	25.87	13.79	11.31	23.74	80.54	33.13	47.56
13								
14	9.93	8.43	22.08	6.51	13.03	50.30	15.86	12.62
15	12.61	22.64	45.74	43.12	64.61	61.85	32.31	35.43

Parameter: Syn parameters						
	Syn num			walk-DMC		
ID	Normal	AFO	KAFO	Normal	AFO	KAFO
1	3	3	4	82.02	92.63	94.41
2	4	3	4	106.99	95.90	102.51
3	4	4		94.57	86.12	
4	4	4		103.92	106.69	
5	5	4	4	120.15	115.20	122.31
6	5	4	4	97.39	87.91	87.75
7	4	4	3	97.18	90.39	82.01
8	4	4	4	95.41	98.69	92.28
9	4	3	3	92.86	94.05	89.07
10	3	4	3	102.07	98.03	95.59
11	4	3	4	101.14	98.88	108.39
12	4	4	3	99.69	108.30	91.50
13	3	4		87.45	87.52	
14	6	4	4	98.54	101.56	105.14
15	4	4	4	120.62	105.44	105.46
